# Pathological Gambling: A Neurobiological Approach Through a Literature Review

**DOI:** 10.1192/j.eurpsy.2025.996

**Published:** 2025-08-26

**Authors:** N. Sghaier, A. Ben Haouala, I. Zariat, N. Romdhane, H. Ben Garouia

**Affiliations:** 1 Psychiatry Departement, Ibn Jazzar Hospital, Kairouan; 2 Psychiatry Departement; 3 Child Psychiatry Departement, Fattouma Bourguiba Hospital, Monastir, Tunisia

## Abstract

**Introduction:**

Gambling is an increasingly widespread practice worldwide. Currently, gambling disorder (also known as pathological gambling) is recognized as a behavioral addiction in the DSM-5 due to its numerous similarities with substance addiction. Consequently, several neurobiological hypotheses have been tested in recent years.

**Objectives:**

To illustrate, through a literature review, the neurobiological basis of pathological gambling.

**Methods:**

We conducted a systematic review of the literature in the “PubMed” database, following PRISMA guidelines, using the following keywords: “Neurobiology,” “Gambling Disorder,” “Pathological Gambling,” and “Gambling.”

**Results:**

Our study included 27 articles, comprising 15 articles addressing pathological gambling from a neuroimaging perspective, 7 articles focusing on neurochemistry, and 5 articles discussing the therapeutic implications of neurobiological mechanisms. Literature studies reveal that the primary neurobiological mechanisms explaining pathological gambling involve dysfunctions in the brain circuits of the “reward system,” particularly in the striatum and ventromedial prefrontal cortex. The studies also highlighted the central role of dopamine and dopaminergic receptors, as well as the involvement of other noradrenergic, serotonergic, glutamatergic, and opioid systems in the disorder’s development, with preliminary evidence suggesting the effectiveness of medications that alter these neurotransmitters.

**Conclusions:**

Pathological gambling is an increasingly common psychiatric disorder that remains underestimated to this day. Therefore, it is important to clarify the neurobiological mechanisms involved in the etiology of this disorder to develop targeted intervention strategies.

**Disclosure of Interest:**

None Declared

